# Fecal Calprotectin and C-Reactive Protein Association With Histologic and Endoscopic Endpoints in Mirikizumab-Treated Patients With Ulcerative Colitis

**DOI:** 10.1093/crocol/otaf043

**Published:** 2025-06-14

**Authors:** Remo Panaccione, Faye Chan-Diehl, Simin Baygani, Deborah A Fisher, Richard E Moses, Britta Siegmund, Alissa Walsh, Taku Kobayashi, Parambir S Dulai, Simon Travis

**Affiliations:** Division of Gastroenterology and Hepatology, University of Calgary, Calgary, Canada; Eli Lilly and Company, Indianapolis, IN, USA; Eli Lilly and Company, Indianapolis, IN, USA; Eli Lilly and Company, Indianapolis, IN, USA; Eli Lilly and Company, Indianapolis, IN, USA; Department of Gastroenterology, Infectious Diseases, and Rheumatology, Universitätsmedizin Berlin, Corporate Member of Freie Universität Berlin and Humboldt-Universität zu Berlin, Berlin, Germany; Translational Gastroenterology Unit, Oxford Biomedical Research Centre and John Radcliffe Hospital, Oxford, UK; Center for Advanced IBD Research and Treatment, Kitasato University Kitasato Institute Hospital, Tokyo, Japan; Department of Medicine (Gastroenterology and Hepatology), Northwestern University, Chicago, IL, USA; Translational Gastroenterology Unit, Oxford Biomedical Research Centre and John Radcliffe Hospital, Oxford, UK; Kennedy Institute, University of Oxford, Oxford, UK

**Keywords:** ulcerative colitis, CRP, C-reactive protein, fecal calprotectin, mirikizumab

## Abstract

**Background:**

Fecal calprotectin (FC) and C-reactive protein (CRP) are noninvasive biomarkers used in ulcerative colitis (UC) clinical trials; however, thresholds defined as “normal” in trials may be higher than “normal” thresholds typically used in clinical practice. We assessed the relationship between FC and CRP improvement in the “normal” range across different cutoff thresholds for patients with moderately to severely active UC treated with mirikizumab.

**Methods:**

Patients achieving clinical response to mirikizumab in LUCENT-1 (Weeks 0-12) proceeded to LUCENT-2 (Weeks 12-52 [52 weeks of continuous mirikizumab]). Associations between FC and CRP levels at multiple thresholds and histologic-endoscopic mucosal improvement (HEMI) and histologic-endoscopic mucosal remission (HEMR) at Weeks 12 and 52 were assessed by Fisher’s exact test. Least squares means of FC and CRP changes from baseline at Weeks 12 and 52 were calculated using analysis of covariance with HEMI or HEMR status as factors and baseline FC or CRP values as covariates.

**Results:**

At Weeks 12 and 52, greater proportions of patients with FC thresholds of ≤250, ≤150, ≤100, and ≤50 µg/g, and CRP thresholds of ≤6 and ≤5 mg/L, achieved HEMI and HEMR compared with those not achieving HEMI and HEMR. Changes from baseline in FC and CRP at Week 12 and FC at Week 52 were greater in patients who achieved HEMI and HEMR compared with those not achieving these endpoints.

**Conclusions:**

These results show that FC and CRP analyses may contribute to a noninvasive monitoring strategy in clinical practice.

ClinicalTrials.gov numbers: NCT03518086, NCT03524092.

## Graphical Abstract

**Graphical Abstract:**
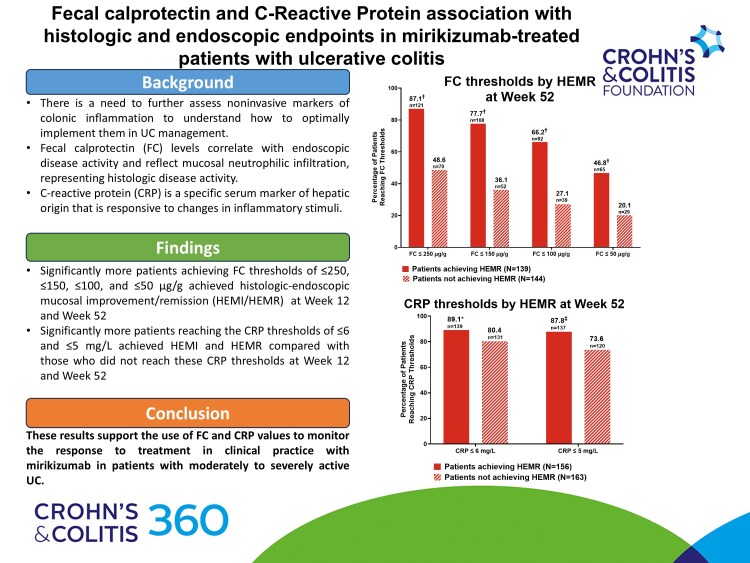

## Introduction

Ulcerative colitis (UC) is a chronic immune-mediated disease characterized by mucosal inflammation of the colon. Treatment targets in UC include symptomatic and endoscopic remission as well as normalization of serum and fecal biomarkers.^1^ Histologic healing is an evolving target as it has been associated with improved clinical outcomes.^2^ Although endoscopic and histologic targets are important, repeated endoscopic evaluation and obtaining biopsies are burdensome to patients. Therefore, there is a need to better characterize the accuracy of noninvasive markers of colonic inflammation and to understand how to optimally implement them in UC management.^3^ Analyses of fecal and serum biomarkers are a noninvasive strategy to monitor disease severity and improvement.^3–5^ Fecal calprotectin (FC) levels correlate with endoscopic disease activity and reflect mucosal neutrophilic infiltration, representing histologic disease activity.^6,7^ Fecal calprotectin levels may indicate treatment response earlier than endoscopy.^8^ C-reactive protein (CRP) is a specific serum marker of hepatic origin that is responsive to changes in inflammatory stimuli.^9^ The combination of FC with clinical activity indices or CRP may improve the prediction of endoscopic active disease or remission in these patients. Fecal calprotectin and CRP cutoff values used to define “normal” for clinical trial endpoints may be higher than typical values defined as “normal” in clinical practice.^10^ These “normal” threshold values should be characterized for clinical treatment decisions.^11,12^

Mirikizumab is an anti-interleukin-23 p19 subunit-targeted antibody that has demonstrated efficacy and safety in moderately to severely active UC.^13^ The aim of this study was to assess the relationship between FC and CRP values across different cutoff thresholds used in clinical practice with histologic-endoscopic mucosal improvement (HEMI) and histologic-endoscopic mucosal remission (HEMR), using data from the LUCENT-1 and LUCENT-2 trials (NCT03518086, NCT03524092).

## Methods

### Ethical Considerations

All patients were required to provide informed consent for participation in the study. The protocol, amendments, and consent documentation were approved by local ethical review boards. The study was registered at the European Network of Centers for Pharmacoepidemiology and Pharmacovigilance and was conducted according to the International Council for Harmonisation of Technical Requirements for Pharmaceuticals for Human Use guidelines, including Good Clinical Practices and Good Pharmacoepidemiology Practices, as well as the Declaration of Helsinki. An independent Data Monitoring Committee monitored LUCENT-1 and LUCENT-2.

### Study Design and Participants

**Figure 1.**
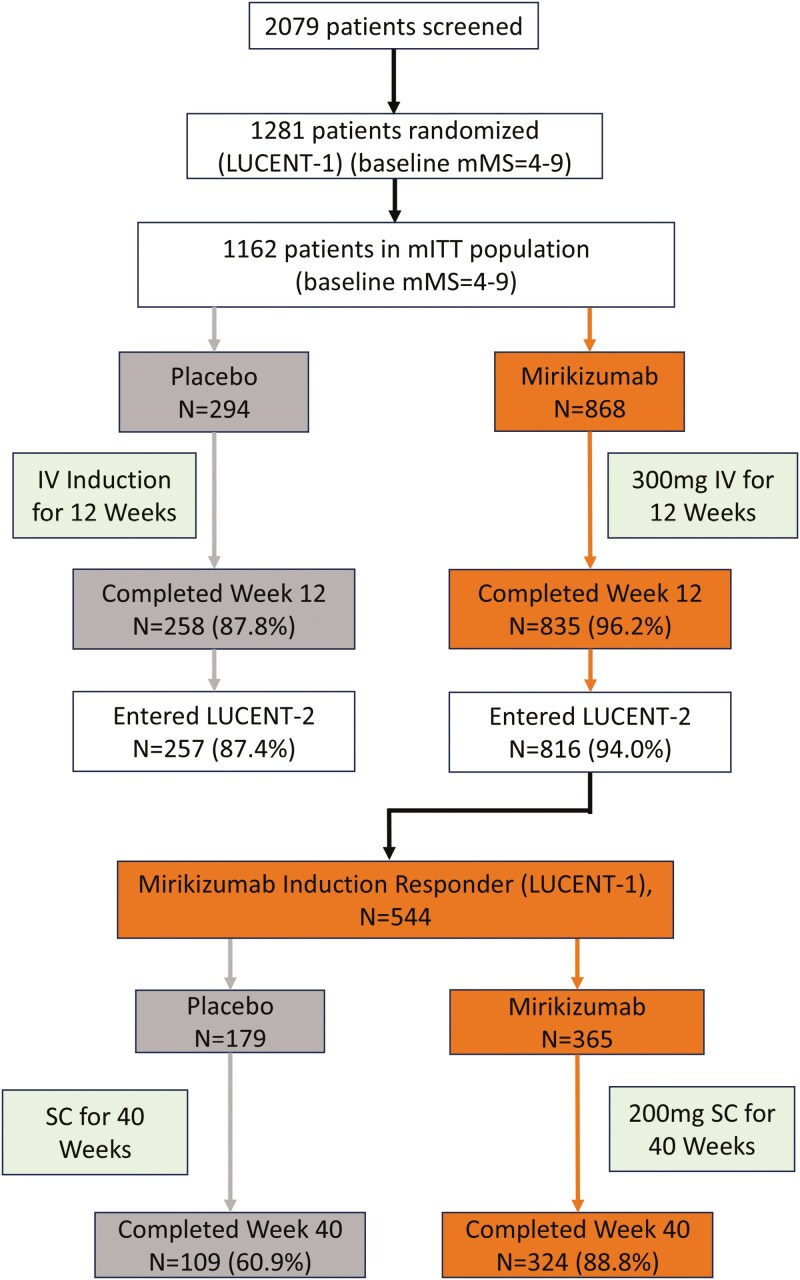
The LUCENT clinical trial program. Abbreviations: IV, intravenous; mITT, modified intent-to-treat; mMS, modified Mayo Score; *N*, number of patients in analysis group; SC, subcutaneous.

Detailed methods for the LUCENT-1 and LUCENT-2 trials have been published previously.^13^Figure 1 summarizes the patient dispositions of the LUCENT clinical trial program and highlights the LUCENT-2 mirikizumab responder population analyzed in the present study. All authors had access to the study data and reviewed and approved the final manuscript.

In the LUCENT-1 blinded induction study (Weeks 0 to 12), patients (18 to 80 years of age) had an established diagnosis of moderately to severely active UC (modified Mayo Score of 4 to 9) for at least 3 months (*N* = 1162). Patients achieving clinical response to mirikizumab at Week 12 proceeded to the LUCENT-2 blinded randomized withdrawal maintenance study (Weeks 12 to 52).^13^

### Randomization and Masking

LUCENT-1 patients were randomized 3:1 to receive intravenous (IV) 300 mg mirikizumab every 4 weeks (Q4W) or placebo. In LUCENT-2, LUCENT-1 Week 12 mirikizumab responders were re-randomized 2:1 to subcutaneous (SC) mirikizumab 200 mg or placebo. In this manuscript the weeks of treatment were cumulative, for example, Week 52 of treatment corresponded to 40 weeks of the LUCENT-2 maintenance trial.

### Procedures

Endoscopy with biopsies was performed at screening, Week 12, and Week 52. Blinded central readers evaluated the endoscopic and histologic endpoints. Biomarkers were collected at Weeks 0, 4, 12, 24, 36, 48, and 52.

### Outcome Measures

HEMI was defined as a Mayo endoscopic subscore of 0 or 1 (excluding friability) and Geboes score of less than or equal to 3.1. HEMR was defined as an endoscopic subscore of 0 or 1 (excluding friability) and Geboes score of less than or equal to 2B.0.^13^

### C-Reactive Protein and Fecal Calprotectin Assessment

Previous LUCENT study reports defined normal CRP as ≤6 mg/L and FC as ≤250 μg/g.^13^ Nonetheless, clinical trial thresholds may differ from clinical practice. The American Gastroenterological Association (AGA) Clinical Practice Guideline notes that a CRP of <5 mg/L and FC levels of <250, <150, or <50 μg/g are typically used in practice.^3^ Recommendations from the AGA describe that an FC <150 μg/g can rule out active inflammation and that a threshold of <50 μg/g may be desired for a patient in symptomatic remission after therapy.^3^ The Selecting Therapeutic Targets in Inflammatory Bowel Disease update (STRIDE-II) proposes a cutoff of <250 μg/g representing minimal to no endoscopic inflammation and <100 μg/g reflecting deeper healing (eg, histologic).^1^ In total we examined all thresholds that appeared in previous studies, are commonly used in practice, or are included in recommendations: CRP of <6 and <5 mg/L; FC <250, <150, <100, and <50 μg/g.

### Statistical Analysis

The LUCENT-1 modified intent-to-treat analysis population included 868 patients treated with mirikizumab IV Q4W. The LUCENT-2 analysis population included LUCENT-1 Week 12 mirikizumab induction responders re-randomized to mirikizumab SC Q4W (*N* = 365) for up to 40 weeks of maintenance therapy (52 weeks of continuous treatment).

Associations between improvement in FC levels at thresholds of ≤250, ≤150, ≤100, and ≤50 µg/g, and achievement of HEMI and HEMR at Week 12 and Week 52 were assessed by Fisher’s exact test. Similarly, associations between improvement in CRP levels at thresholds of ≤5 and ≤6 mg/L and achievement of HEMI and HEMR at Week 12 and Week 52 were assessed by Fisher’s exact test. Least squares means of FC and CRP changes from baseline in FC and CRP levels at Week 12 and Week 52 were calculated using an analysis of covariance (ANCOVA) with HEMI or HEMR status as a factor and baseline FC or CRP value as a covariate. Patients that were missing any of these data points at baseline and Weeks 12 and 52 were not included in the analysis, and no missing data imputation was used. Patients in the extended induction cohort were not included in this analysis.

## Results

Of the 868 patients randomized to mirikizumab induction, 544 patients achieved clinical response, and 365 patients were re-randomized to mirikizumab for maintenance. Baseline demographic and disease characteristics for the LUCENT-1 mirikizumab induction group and the LUCENT-2 mirikizumab maintenance group are presented in Table 1. Mean baseline FC levels were 3134.2 and 2880.0 µg/g in the LUCENT-1 mirikizumab induction group and LUCENT-2 mirikizumab maintenance group, respectively; the mean baseline CRP levels were 9.3 and 8.9 mg/L, respectively (Table 1).

**Table 1.** Baseline demographic and disease characteristics (mITT population).

	Mirikizumab induction (LUCENT-1, *N* = 868)	Responders to mirikizumab induction re-randomized to mirikizumab maintenance (LUCENT-2, *N* = 365)
**Age, years, mean (SD)**	42.9 (13.9)	43.4 (14.2)
**Male, *n* (%)**	530 (61.1)	214 (58.6)
**Race, *n* (%)**		
** American Indian or Alaska Native**	10 (1.2)	3 (0.8)
** Asian**	223 (26.0)	93 (25.6)
** Black or African American**	10 (1.2)	6 (1.7)
** Native Hawaiian or Other Pacific Islander**	1 (0.1)	0
** White**	614 (71.5)	261 (71.9)
** Multiple**	1 (0.1)	0
**Duration of UC, years, mean (SD)**	7.2 (6.7)	6.9 (7.1)
**Baseline disease location, *n* (%)**		
** Proctitis**	6 (0.7)	3 (0.8)
** Left-sided colitis**	544 (62.7)	234 (64.1)
** Pancolitis**	318 (36.6)	128 (35.1)
**MMS category, *n* (%)**		
** Mild (1–3)**	1 (0.1)	0
** Moderate (4–6)**	404 (46.5)	181 (49.6)
** Severe (7–9)**	463 (53.3)	184 (50.4)
**FC, µg/g, mean (SD)** ^*^	3134.2 (4846.6)	2880.0 (4620.8)
**CRP, mg/L, mean (SD)** ^*^	9.3 (14.7)	8.9 (14.7)
**Prior biologic or tofacitinib failure, *n* (%)**	361 (41.6)	128 (35.1)

Abbreviations: CRP, C-reactive protein; FC, fecal calprotectin; mITT, modified intent-to-treat; MMS, modified Mayo Score; *n*, sample size; *N*, number of patients in analysis group; SD, standard deviation; UC, ulcerative colitis.

^*^Among the mirikizumab induction group (*N* = 868), 766 and 828 patients had available data for baseline FC and CRP values, respectively. Among responders to mirikizumab induction re-randomized to mirikizumab maintenance (*N* = 365), 283 and 319 patients had available data for LUCENT-1 baseline FC and CRP values, respectively. Study participants who were missing values for FC and/or CRP were excluded from subsequent analyses.

**Figure 2.**
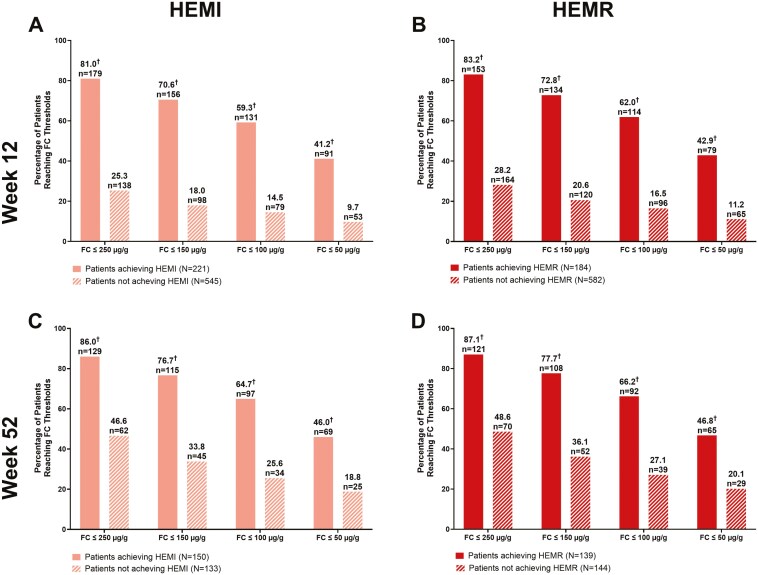
Proportions of patients reaching FC thresholds who also reached (A) Week 12 HEMI, (B) Week 12 HEMR, (C) Week 52 HEMI, and (D) Week 52 HEMR. Abbreviations: FC, fecal calprotectin; HEMI, histologic-endoscopic mucosal improvement; HEMR, histologic-endoscopic mucosal remission; *N*, number of patients in analysis group. ^†^*P* < .0001 for the proportion of patients with FC at threshold or below achieving HEMI or HEMR versus not achieving HEMI or HEMR. *P*-value is from Fisher’s exact test.

Significantly greater proportions of patients achieving FC thresholds of 250 µg/g or less, 150 µg/g or less, 100 µg/g or less, and 50 µg/g or less also achieved HEMI and HEMR compared with those who did not reach FC thresholds at Week 12 and Week 52 (Figure 2).

**Figure 3.**
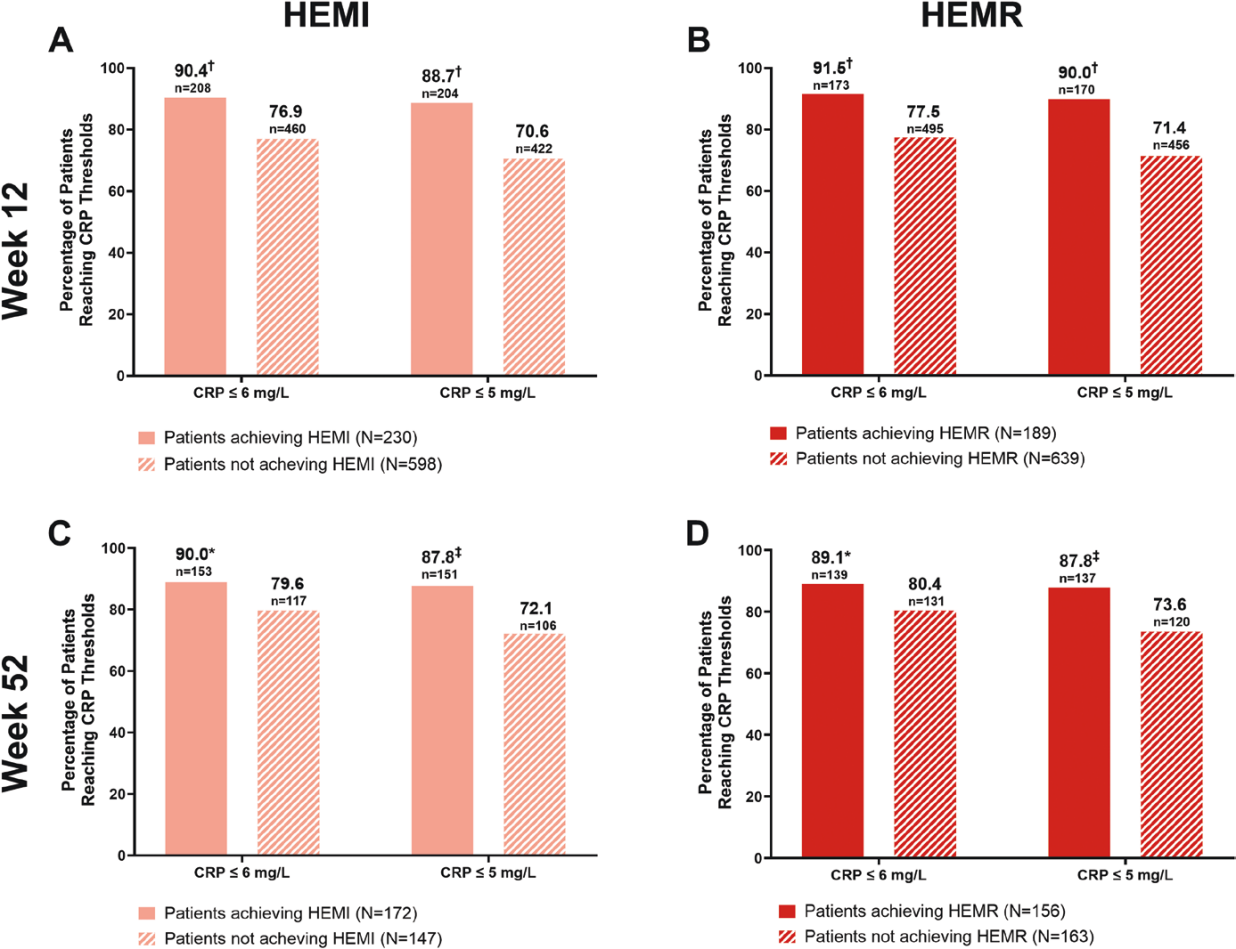
Proportions of patients reaching CRP thresholds who also reached. (A) Week 12 HEMI, (B) Week 12 HEMR, (C) Week 52 HEMI, and (D) Week 52 HEMR. Abbreviations: CRP, C-reactive protein; HEMI, histologic-endoscopic mucosal improvement; HEMR, histologic-endoscopic mucosal remission; *N*, number of patients in analysis group. *P < .05, ^‡^*P* < .01, ^†^*P* < .0001 for the proportion of patients with CRP at threshold or below achieving HEMI or HEMR versus not achieving HEMI or HEMR. *P*-value is from Fisher’s exact test.

**Figure 4.**
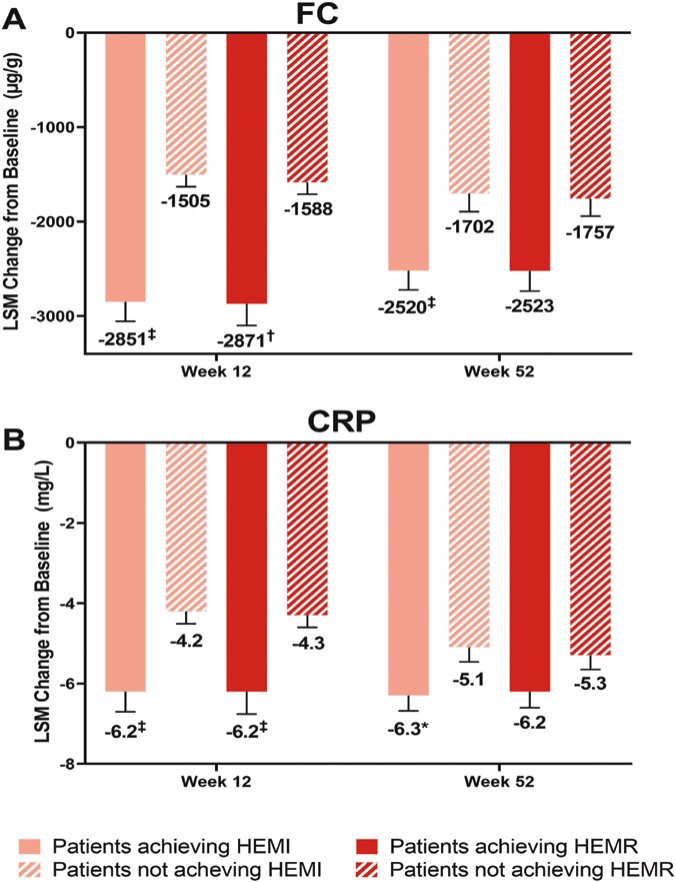
Changes from baseline in (A) FC and (B) CRP by HEMI and HEMR achievement at Week 12 and Week 52. Abbreviations: ANCOVA, analysis of covariance; CRP, C-reactive protein; FC, fecal calprotectin; HEMI, histologic-endoscopic mucosal improvement; HEMR, histologic-endoscopic mucosal remission; LSM, least squares mean. *P*-value is from ANCOVA with HEMI or HEMR status as a factor and baseline FC or CRP value as a covariate. *P < .05, ^‡^*P* < .01, ^†^*P* < .0001 for least squares mean difference in patients achieving HEMI or HEMR versus not achieving HEMI or HEMR.

Significantly greater proportions of patients reaching the CRP thresholds of ≤6 and ≤5 mg/L achieved HEMI and HEMR compared with those who did not reach these CRP thresholds at both Week 12 and Week 52 (Figure 3). At both Week 12 and Week 52, changes from baseline in FC and CRP levels were greater in magnitude in patients who achieved HEMI or HEMR than in those who did not achieve these histologic-endoscopic endpoints (Figure 4). Additionally, patients who were corticosteroid-free through induction showed 41.5% clinical remission at Week 52 (Supplementary Figure S1).

## Discussion

Biomarkers are used frequently for noninvasive monitoring and treatment decisions in the management of patients with UC. The 2023 AGA guidelines on the role of biomarkers for UC management have recommended a monitoring strategy based on both symptoms and biomarkers. For patients achieving symptomatic remission, the AGA suggests excluding active disease using FC of less than 150 µg/g, and/or “normal” CRP (<5 mg/L), in lieu of repeated endoscopies.^3^ Additionally, a stricter cutoff for FC of 50 µg/g or less could be preferable in patients who achieve symptomatic remission after a recent treatment adjustment. In patients who have achieved symptomatic remission, CRP normalization could suggest endoscopic response if CRP was elevated when the patient was experiencing active disease.^3^ The STRIDE-II has proposed FC levels between 100 and 250 µg/g and a CRP threshold of <5 mg/L as UC treatment targets.^1^ A threshold of 250 µg/g or less can reflect a Mayo endoscopic subscore of 0 or 1, and lower thresholds of 100 µg/g or less may serve as a marker of “deeper” endoscopic or histologic healing.^1^ Interestingly, location and extent of disease have not been shown to influence the degree of endoscopic activity. In total, the threshold levels assessed in this report provide results that are clinically relevant. Our results show that FCP thresholds used in clinical trials as well as FCP levels used in clinical practice are associated with achievement of HEMI and HEMR.

Clinical trials are necessary to establish the efficacy and safety of a drug; however, clinical trial endpoints and study designs differ from clinical practice in important ways. For clinical trials in UC, these differences include performing endoscopy at the end of induction, which is often not routine in clinical practice, and using higher cutoff values for FC and CRP endpoints, for example, 250 µg/g and 6 mg/dL, respectively. In this analysis, we have provided clinically actionable information by examining several thresholds of FC and CRP, including more stringent cutoff values referenced in guidelines^1,3^ and those commonly used in clinical practice.^8,9^ We analyzed biomarker thresholds at the end of induction, when endoscopy is not generally performed, although this is a point in UC management when an objective measure of inflammation facilitates maintenance therapy decisions. We also analyzed biomarker thresholds at the end of 40 weeks of maintenance therapy (52 weeks of continuous treatment).

For patients who responded to mirikizumab induction and were re-randomized to mirikizumab maintenance, there were significantly more patients reaching FC and CRP thresholds at Week 12 and Week 52 who achieved HEMI and HEMR compared with those who did not achieve HEMI or HEMR. This was demonstrated across all FC and CRP threshold levels tested. The association between biomarkers and histologic-endoscopic endpoints was more pronounced with FC than CRP at both timepoints (Figures 2 and 3). Although not seen in CRP, the reason HEMI/HEMR seems to decrease with higher thresholds of FC is currently unclear although this could possibly be due to sample size changes or continued low-grade microscopic inflammation.

These results indicate that the noninvasive biomarkers, FC and CRP, can be part of a strategy in clinical practice to noninvasively monitor histologic and endoscopic outcomes in patients with moderately to severely active UC treated with mirikizumab, as has been suggested.^1,3^

Our results are consistent with previously published results. A post hoc analysis of data from the GEMINI 1/LTS and VARSITY trials evaluating vedolizumab and adalimumab for treatment of moderately to severely active UC reported that an FC threshold of 250 µg/g or less was associated with an increased likelihood of achieving clinical, endoscopic, and histologic endpoints at Week 52, as well as a lower risk of long-term UC-related complications such as colectomy and hospitalization.^8^ Another post hoc analysis of data from the phase 4 MOMENTUM trial evaluating multimatrix mesalamine for mildly to moderately active UC reported that median FC concentrations were lower in patients who achieved clinical, endoscopic, and histologic endpoints compared with those who did not, and that optimal FC thresholds for identifying histologic remission at Weeks 8 and 52 were 75 and 99 µg/g, respectively.^5^

The accuracy, lower cost, and relative ease of FC and CRP evaluation compared with endoscopy make them attractive options for monitoring UC disease activity^1^; however, FC and CRP have limitations in some clinical contexts. Fecal calprotectin may be elevated due to many luminal inflammatory processes, including infection, and CRP may be elevated in patients due to non-intestinal sources of infection or inflammation. In patients with mild UC, FC and CRP may not be useful in detecting endoscopic or histologic remission or mild endoscopic disease activity.^3^ Biomarkers may not correlate with endoscopic disease activity in some patients as FC and CRP levels may be dependent on disease extent and location.^3,6,14^ Patient adherence in providing stool samples may be an additional barrier to the use of FC for monitoring disease.^15^ The use of varying cutoff thresholds for FC and the lack of consensus on definitions of disease activity, including endoscopic and histologic remission, also complicate the use of biomarkers in UC disease monitoring and management.^5,7^

Our study has limitations inherent to post hoc analyses. The a priori clinical trial definitions in LUCENT-1 and LUCENT-2 for “normalization” were FC ≤250 µg/g and CRP ≤6 mg/L.^13^ Our multiple threshold analysis did not have sufficient sample size to account for prior biologic use. In the primary clinical trial analyses,^13^ a higher percentage of biologic- and tofacitinib-naïve patients treated with mirikizumab achieved HEMI at Week 12 and HEMR at Week 52 compared with mirikizumab-treated patients who had previously experienced treatment failure with a biologic or tofacitinib.^13^ The current analyses cannot comment on the relationship between FC and CRP and histologic-endoscopic outcomes after treatment with other drugs, although our results are consistent with studies of other UC treatments. Finally, although receiver operating characteristic curves can be used to assess the overall diagnostic performance of two or more diagnostic tests, in this report it was determined that they were not as clinically useful compared with other described analyses.

Strengths of this evaluation include the use of phase 3 clinical trial data from a large patient population who has had consistent treatment with mirikizumab through Week 52. Our study provides data on the relationship between the biomarkers FC and CRP, and HEMI/HEMR at the end of induction and after 52 weeks of treatment across a range of thresholds used in clinical practice rather than solely in clinical trials. This supports recent recommendations to utilize noninvasive biomarkers in the management of UC and will be useful for clinicians managing UC patients.

## Conclusions

These results support the use of FC and CRP values to monitor the response to treatment in clinical practice with mirikizumab in patients with moderately to severely active UC. This patient-centric strategy is consistent with current guidelines and facilitates management decisions based on objective measures of inflammation that are available earlier and are more feasible than repeat endoscopies and biopsies.

## Supplementary Material

otaf043_suppl_Supplementary_Figure_S1

## Data Availability

Data are available on reasonable request. Eli Lilly and Company provides access to all individual participant data collected during the trial after anonymization. Data are available to request after primary publication acceptance. No expiration date of data requests is currently set once data are made available. Access is provided after a proposal has been approved by an independent review committee identified for this purpose and after receipt of a signed data-sharing agreement. Data and documents, including the study protocol, statistical analysis plan, clinical study report, and blank or annotated case report forms, will be provided in a secure data-sharing environment. For details on submitting a request, see the instructions provided at www.vivli.org.
