# Supplementary material for: Fecal Calprotectin and C-Reactive Protein Association With Histologic and Endoscopic Endpoints in Mirikizumab-Treated Patients With Ulcerative Colitis
Source: Crohns Colitis 360. 2025 Jun 14;7(2):otaf043. doi: 10.1093/crocol/otaf043 (PMC12207289; doi:10.1093/crocol/otaf043)
Supplement: otaf043_suppl_Supplementary_Figure_S1 [file otaf043_suppl_supplementary_figure_s1.docx]

**Supplemental Figure 1. Proportions of patients achieving CS-free remission at W52.**

CS, corticosteroid; NRI, non-responder imputation. *p≤0.001

CS-free remission at Week 52 was defined as clinical remission at Week 52 + remission of symptoms at Week 40, and no CS use for at least 12 weeks before Week 52.
